# Comparative Efficacy of a Novel Topical Formulation with Antimicrobial Peptides and Encapsulated Plant Extracts Versus Conventional Therapies for Canine Otitis Externa

**DOI:** 10.3390/pathogens14111112

**Published:** 2025-11-01

**Authors:** Tatiana Charello Bannach, Anna Claudia Baumel Mongruel, Alberto Gonçalves Evangelista, Vitória Brigida Mielnik de Souza, Renata Voi, Michel Fleith Otuki, Marconi Rodrigues de Farias, Fernando Bittencourt Luciano

**Affiliations:** 1 Graduate Program in Animal Science, Pontifícia Universidade Católica do Paraná (PUCPR), Curitiba 80215-901, PR, Brazil; 2 Laboratory of Microbiology Research, Biopark Educaçäo, Toledo 85919-899, PR, Brazil; alberto.evangelista@bpkedu.com.br; 3 Department of Pharmacology, Universidade Federal do Paraná, Curitiba 81531-980, PR, Brazil

**Keywords:** atopic dermatitis, dermatology, antimicrobial resistance, veterinary

## Abstract

Canine otitis externa (OE) presents a significant challenge in veterinary medicine due to its complex, multifactorial nature and the growing issue of antimicrobial resistance (AMR) associated with conventional antibiotic use. The objective of this study was to compare the efficacy of a novel, antibiotic-free topical ear solution (Therapy A) containing antimicrobial peptides and encapsulated plant extracts (chamomile, calendula, rosemary, and hops) against a standard conventional treatment (Therapy B) composed of gentamicin, betamethasone valerate, and clotrimazole. A longitudinal, randomized study was conducted over four weeks with 40 domestic dogs diagnosed with OE. The dogs were divided into two groups, each receiving one of the therapies. Evaluations were performed weekly, assessing clinical signs using the Otitis Index Scoring System (OTIS-3) and a pruritus visual analog scale (pVAS), as well as ear canal pH and cytology. The results showed that Therapy A provided similar clinical efficacy in OTIS-3 and pVAS scores that were comparable to Therapy B. Cytological analysis also revealed a significant reduction in microbial presence for both groups. Notably, Therapy A was clinically effective in two of the three dogs presenting multi-drug resistant (MDR) bacterial infections. The novel formulation also demonstrated a favorable safety profile, with no adverse drug reactions reported, in contrast to one dog in the conventional treatment group that experienced an adverse reaction. These findings suggest that the plant-based formulation is a safe and effective alternative for managing canine OE, offering a promising solution to reduce the reliance on antibiotics and corticosteroids.

## 1. Introduction

Otitis externa (OE) is a common inflammatory condition affecting the external ear canal in dogs, impacting approximately 20% of the canine population [1]. Clinical signs include discomfort, pain, pruritus, malodor, discharge and head shaking [2]. Chronic OE can lead to severe manifestations like ear canal stenosis, hyperkeratosis, lichenification, tympanic membrane rupture, and even progression to otitis interna and hearing loss [3]. The disease is often complicated by dysbiosis and secondary bacterial or fungal infections often accompany the inflammatory process, with recurrent episodes commonly linked to antimicrobial resistance (AMR) and biofilm formation due to microbial overgrowth [4].

The etiology of OE is multifactorial. Primary causes include parasites, hypersensitivity disorders (particularly atopic dermatitis), immune-mediated conditions and keratinization or endocrine disorders [5]. Chronic OE is particularly associated with allergic skin diseases, such as atopic dermatitis (AD), which affects 10–15% of dogs and is a common trigger for OE development [6]. Secondary bacterial and fungal overgrowth, most frequently involving *Staphylococcus pseudintermedius*, *Pseudomonas* spp., *Proteus* spp., and *Malassezia* spp., is frequently reported [7]. These microorganisms, while commensal at low levels, may proliferate under inflammatory conditions, amplifying tissue damage and discomfort. Chronic OE, especially when associated with allergic skin diseases, remains one of the most challenging presentations to manage in veterinary dermatology routine [8].

Current treatment protocols often rely on topical formulations combining antibiotics, antifungals and corticosteroids to reduce microbial load and inflammation [9]. However, the extensive and repeated use of antibiotics has favored the selection of resistant strains, particularly multidrug-resistant *Staphylococcus* spp. and *Pseudomonas* spp. [10]. This not only complicates local disease management but also raises concerns about zoonotic transmission of resistant organisms [10,11,12,13]. The search for alternative topical therapies that can control infection and inflammation while reducing the selective pressure for AMR is, therefore, a priority.

Plant-derived compounds, such as chamomile (*Matricaria chamomilla*), marigold (*Calendula officinalis*), rosemary (*Rosmarinus officinalis*) and hops (*Humulus lupulus*), possess well-documented anti-inflammatory, antimicrobial and wound-healing properties that may be relevant to the management of OE [14,15,16,17]. In parallel, antimicrobial peptides (AMPs) produced by keratinocytes and commensal skin microbiota exert broad-spectrum activity against bacteria and fungi and play an important role in immune regulation [18,19,20]. The combination of plant-derived bioactive extracts with AMPs may, therefore, represent a promising approach for reducing pathogen burden, alleviating inflammation and supporting tissue repair in canine OE.

Based on these considerations, the aim of this study was to evaluate whether an ear solution containing antimicrobial peptides and encapsulated plant extracts as chamomile, marigold, rosemary and hops (Therapy A) provides clinical efficacy comparable to a conventional otic formulation containing gentamicin, betamethasone valerate, clotrimazole (Therapy B), while potentially reducing the need for antibiotics and corticosteroids in dogs with otitis externa.

## 2. Materials and Methods

### 2.1. Study Design and Ethics Statement

A longitudinal, simple randomized study was conducted with 40 domestic dogs diagnosed with OE. Dogs received veterinary care at the University Veterinary Clinic of the Pontifical Catholic University of Paraná (PUCPR) in Curitiba or a private veterinary hospital in Ponta Grossa. The study was approved by the Ethics Committee for Animal Use at PUCPR (protocol 5684230523). Informed client consent was obtained via signature upon enrollment.

Dogs diagnosed with bacterial and/or fungal otitis externa, regardless of the clinical signs observed, were included. Additional criteria required a minimum age of 12 months and preservation of the tympanic membrane, confirmed by otoscopic examination performed by a trained veterinary dermatologist.

Exclusion criteria included severe systemic illness, dermatological and systemic comorbidities precluding treatment, pregnancy, continuous antipruritic medications (oclacitinib, lokivetmab, topical or systemic glucocorticoids), systemic antimicrobial therapy or topical antiseptics within 30 days prior, tympanic membrane perforation, or ear canal neoplasms.

Group allocation was randomized. Group A (GA) received exclusive treatment with a new ear solution containing antimicrobial peptides, encapsulated chamomile (*Matricaria chamomilla*), marigold (*Calendula officinalis*), rosemary (*Rosmarinus officinalis*) and hops (*Humulus lupulus*) extracts (Wesen Green Solutions^®^, São Paulo, SP, Brazil) every 12 h for 28 d; group B (GB) received exclusive treatment with an ear solution containing 0.3% of gentamicin, 0.122% of betamethasone valerate, and 1% of micronized clotrimazole (Otomax, MSD Animal Health^®^, São Paulo, SP, Brazil) every 12 h for 28 d. Each group started with 20 dogs. One dog from GA was excluded due to lack of owner adherence, and one from GB due to drug eruption (final *n* = 38). Topical application of products was performed by the owners, ensuring complete coverage of the ear canal, followed by proper massaging as instructed by the veterinarian. Owners were also advised not to apply any additional products to the ear canal during the study. Owners and evaluators were not blinded to the treatment allocation because the two formulations had distinct characteristics (odor, color, and packaging), making blinding impractical.

Animals were evaluated weekly over four weeks (T0, T7, T14, T21, T28). Safety evaluation involved weekly assessment for erythema, pruritus, abrasions, crusts, and owner reports of restlessness, pain, increased clinical signs, aversion to therapy, excessive licking, sialorrhea, mucosal damage, or vomiting. Animals showing these signs were removed and treated. No systemic blood tests or laboratory parameters were performed as part of the safety monitoring, and adverse effects were assessed solely through clinical observation.

### 2.2. Diagnosis and OTIS-3

The diagnosis of otitis externa was determined through clinical evaluation conducted by a certified veterinary clinician, and infections were confirmed by cytological examination and bacterial culture. The Otitis Index Scoring System (OTIS-3) [21] was employed to assess the severity of otitis. Briefly, OTIS-3 criteria involve four parameters (swelling, erythema, ulceration, and exudate) that were scored on a scale from 0 (absent) to 3 (very severe). The scores for each parameter were then summed up, resulting in a total OTIS-3 score ranging from 0 to 12. OTIS-3 scores were evaluated weekly for all animals. 

### 2.3. Pruritus Evaluation

Ear pruritus was assessed weekly using a visual analog scale (pVAS) (0 = no itching, 10 = severe itching) [22,23]. Owners rated their dog’s ear-specific pruritus, guided by the question, “Has the itch level improved, worsened or stayed the same since the last visit?” [24], with knowledge of the previous pVAS score.

### 2.4. pH Measurement

To assess pH changes in the ear skin microenvironment during treatments, this parameter was measured directly on the pinna internal area using a skin pHmeter (HI 99181, Hanna Instruments^®^, Woonsocket, RI, USA). Measurements were performed weekly.

### 2.5. Cytological Evaluation

Cytological examinations were performed by gently rubbing sterile swabs inside the affected ear canals. The swabs were rotated 3–4 times within the canal to ensure adequate material collection. After sampling, the material was transferred to slides, fixed, and stained using a rapid stain (Panoptic Fast^®^, NewProw, Pinhais, PR, Brazil). The presence of yeasts (*Malassezia* spp.), cocci, or bacilli bacteria in the cytological samples was assessed by visualization using a light microscope (Eclipse E100, Nikon, Tokyo, Japan) (1000×), where 10 different microscope fields were counted, averaged and scored. Yeasts were scaled as follows: 0 (absent–no cells per microscopic field), 1 (rare–up to 2 cells per microscopic field), 2 (moderate–3 to 8 cells per microscopic field), and 3 (abundant–more than 8 cells per microscopic field). Cocci and bacilli bacteria were scored as: 0 (absent–up to 2 cells per microscopic field), 1 (rare–3 to 8 cells per microscopic field), 2 (moderate–9 to 40 cells per microscopic field), and 3 (abundant–more than 40 cells per microscopic field) [25]. Cytological examinations were performed weekly for all animals.

### 2.6. Microbiological Assays

Material from affected ear canals using appropriate sterile swabs at T0 only. After sampling, swabs were immediately stored in Stuart broth and maintained at 4 °C for up to 24 h. Samples were then cultured on Mueller-Hinton agar plates and incubated at 37 °C for 24 h to verify microbial growth and colony morphology. Isolates were re-inoculated on fresh Mueller-Hinton plates, and their proteomic fingerprint was analyzed using Matrix-Assisted Laser Desorption Ionization–Time of Flight Mass Spectrometry (MALDI-TOF MS) (Bruker MALDI Biotyper^®^, Billerica, MA, USA). Antimicrobial susceptibility profiles of 21 isolates from dogs in Curitiba were evaluated via disc diffusion (CLSI guidelines) [26]. Results were categorized as sensitive (S), intermediate (I), or resistant (R). Supplementary File S1 details tested antimicrobials per bacterial group.

### 2.7. Statistical Analysis

Demographic characteristics of the animals included in the study were summarized and presented as percentages. The data included breed, age, sex, sterilization status, and number of ears affected (single or both). The median was calculated for scored parameters (OTIS-3, pVAS, *Malassezia* spp. cells, and bacilli/cocci cells), while the mean was calculated for pH measurements.

For comparisons between groups (GA vs. GB), the Mann–Whitney U test was used to compare all values of a parameter (OTIS-3, pVAS, *Malassezia* spp., bacilli/cocci cells, and pH) at the same time point. Comparisons within the same group across time points were performed using the Wilcoxon signed-rank test. All analyses were conducted using StatsKingdom and RStudio version 4.2.3 software.

## 3. Results

### 3.1. Dogs Included in the Present Study

Initially, 40 dogs were enrolled. One GA dog was removed due to a lack of owner adherence to the treatment. One GB dog was excluded from the study due to the development of an acute drug eruption immediately following the administration of the otological solution and promptly received a different treatment protocol. The final population was 38 dogs (19 GA, 19 GB). The population included 19 females and 19 males (50% each) with a mean age of 5.94 years (range 1–16). Thirteen breeds and mixed-breed dogs were represented. The most frequent were Lhasa Apso (26.32%), Shih Tzu (15.79%), and Golden Retriever (15.79%). Thirty-two (32) dogs had bilateral OE (84.21%), and 6 had unilateral. A total of 70 ears were evaluated, evenly distributed between groups (35 GA, 35 GB). Detailed individual data can be found in Table 1.

### 3.2. Evaluation of Efficacy and pH Evaluation

Efficacy of both treatments was evaluated through OTIS-3 and pVAS scores, alongside cytological analysis and ear skin pH measurements. In Group A (GA), OTIS-3 scores progressively decreased from a median of 7.00 (SE = 0.62) at T0 to 2.00 (SE = 0.46) at T28. Similarly, pVAS scores declined from a median of 8.00 (SE = 0.71) at T0 to 0.00 (SE = 0.39) at T28. Mean ear skin pH in GA ranged from 6.06 (SD = 0.58) at T0 to 6.08 (SD = 0.99) at T28. Cytological analysis revealed a notable reduction in *Malassezia* spp. scores, from a median of 3.00 (SE = 0.15) at T0 to 0.00 (SE = 0.07) at T28. Cocci and bacilli median scores remained at 0.00 throughout the study period.

For Group B (GB), OTIS-3 scores decreased from a median of 7.00 (SE = 1.03) at T0 to 2.00 (SE = 0.50) at T28. pVAS scores also reduced, from 7.00 (SE = 0.47) at T0 to 1.00 (SE = 0.55) at T28. Mean ear skin pH in GB increased from 6.10 (SD = 0.80) at T0 to 6.30 (SD = 1.09) at T28. *Malassezia* spp. scores decreased from a median of 2.00 (SE = 0.50) at T0 to 0.00 (SE = 0.15) at T28. Cocci and bacilli median scores consistently remained at 0.00 from T0 to T28.

Detailed median and mean values for all assessments at each time point are provided in Supplementary File S2. Individual dog data for OTIS-3, pVAS, cell counts, and pH are available in Supplementary File S3.

### 3.3. Microbiological Identification

MALDI-TOF analysis identified 25 bacterial and one fungal (*Malassezia pachydermatis*) species among 60 isolates from 30/38 (78.9%) dogs with OE. The most prevalent microorganisms were *Staphylococcus coagulans* (10/60; 16.7%), *Proteus mirabilis* (9/60; 15%), and *Staphylococcus pseudintermedius* (6/60; 10%).

Fifteen microbial species were isolated from Group A (GA), originating from 16/30 (56.7%) dogs, with *P. mirabilis* being the most prevalent (8/16; 50%). In Group B (GB), 17 species were identified from 14/30 (46.7%) dogs, with *S. coagulans* being the most prevalent (8/17; 47%). Seven species were common to both groups: *S. coagulans, S. pseudintermedius, Pseudomonas aeruginosa, Staphylococcus schleiferi, M. pachydermatis, Bacillus cereus*, and *Escherichia coli*. Additionally, bacteria with unknown/opportunistic pathogenic potential, including *Leclercia adecarboxylata, Paenibacillus nematophilus, Bacillus megaterium, Paenibacillus lautus, Pseudomonas koreensis, Weissella cibaria, Psychrobacter lutiphocae*, and *Bacillus pumillus*, were identified. No colonies were observed in cultured samples from 8/38 (21%) animals. The number of isolates per species is presented in Table 2, and individual animal results are in Supplementary File S4.

Antimicrobial susceptibility testing, performed on 24 re-isolated strains from 14 animals in Curitiba using the disc diffusion method [26], identified three multi-drug resistant (MDR) phenotypes. Two *S. pseudintermedius* isolates (C-05, C-09; both GA) and one *Staphylococcus warneri* isolate (C-01; GA) exhibited resistance to ≥3 antibiotic classes. Specifically, *S. pseudintermedius* from dog C-05 was resistant to β-lactams (oxacillin), tetracyclines (doxycycline), and lincosamides (clindamycin); *P. aeruginosa* was also present in this ear. The *S. pseudintermedius* from dog C-09 was resistant to lincosamides (clindamycin), macrolides (azithromycin), and tetracyclines (doxycycline). The *S. warneri* isolate demonstrated resistance to aminoglycosides (gentamicin), lincosamides (clindamycin), and tetracyclines (doxycycline).

Oxacillin resistance was observed in three staphylococcal isolates: *S. coagulans* (C-03, C-15) and *S. pseudintermedius* (C-05). The *S. pseudintermedius* isolate from C-09 also showed an intermediate inhibition zone for oxacillin, suggesting resistance. Notably, three dogs with bilateral *S. coagulans* infections (C-03, C-08, C-15) exhibited differing susceptibility profiles between ear isolates. For C-03 (GA), the left ear isolate was resistant to cephalexin/oxacillin, while the right was susceptible. For C-08 (GB), the left ear isolate was resistant to azithromycin and intermediately susceptible to cephalexin/oxacillin, while the right was susceptible. For C-15 (GB), the left ear isolate was resistant to cephalexin/oxacillin, while the right was susceptible. Disc diffusion test results for each isolate are presented in Supplementary File S5, and the percentage of resistant isolates for tested antimicrobials is in Supplementary File S6.

### 3.4. Statistical Analysis of Clinical Parameters, Pruritus, pH and Cytology

#### 3.4.1. Comparison of Time Points Between Groups

No significant differences were found in any time point (*p* > 0.05) when comparing GA and GB for OTIS-3, pVAS, pH and cytological scores. All *p*-values obtained in the Mann–Whitney U test are presented in Supplementary File S7.

#### 3.4.2. Comparison of Time Points Within Groups

Both therapies demonstrated time-dependent efficacy, with all surveyed time points significantly different from each other within the same group for OTIS-3 and pVAS (*p* < 0.05). For *Malassezia* spp., both GA and GB showed significant, time-dependent reductions at T7, T14, and T21 (*p* < 0.05), with no further significant difference between T21 and T28 (*p* > 0.05).

Coccoid cell counts in GA significantly decreased at T7 (*p* < 0.05), with no subsequent significant changes (*p* > 0.05). In GB, significant reductions occurred at T7 and T14 (*p* < 0.05), but not thereafter (*p* > 0.05).

Bacilli counts in GA showed no significant changes (*p* > 0.05). Conversely, GB exhibited a significant, time-dependent reduction at T7 and T14 (*p* < 0.05), with no further significant differences (*p* > 0.05).

Ear canal pH remained stable in both groups, with no statistically significant differences observed within or between groups at any time point (*p* > 0.05).

**Figure 1:**
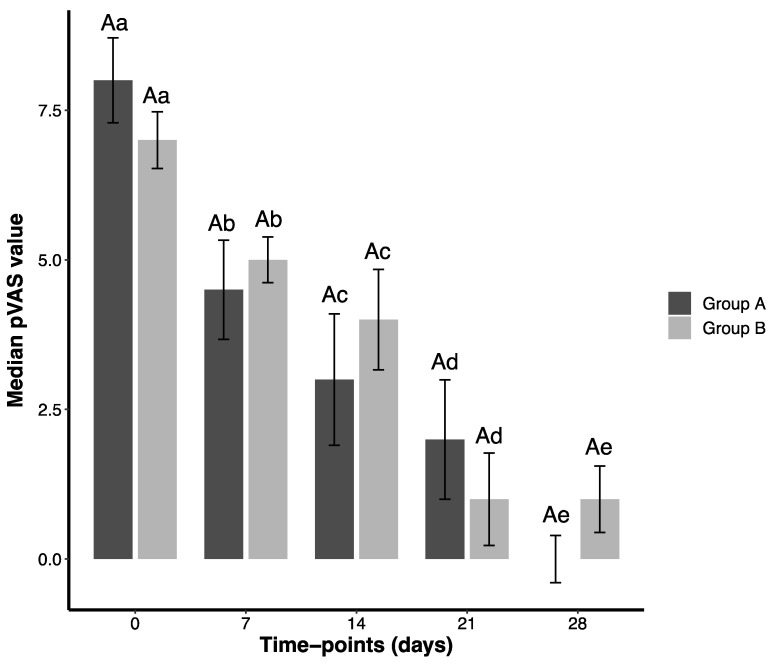
Median values (and standard error) obtained for the pVAS assessment of 38 dogs presenting otitis externa and threatened with different ear solutions during 28 days and evaluated at five time points (T0, T7, T14, T21, T28). Group A (GA) comprised dogs threatened with a natural formula, meanwhile Group B (GB) comprised dogs treated with a conventional medication. Capital letters represent comparisons between groups (GA × GB). Lowercase letters represent comparisons within the same group. Sample size was the same at each time point (35 dogs in each group).

**Figure 2:**
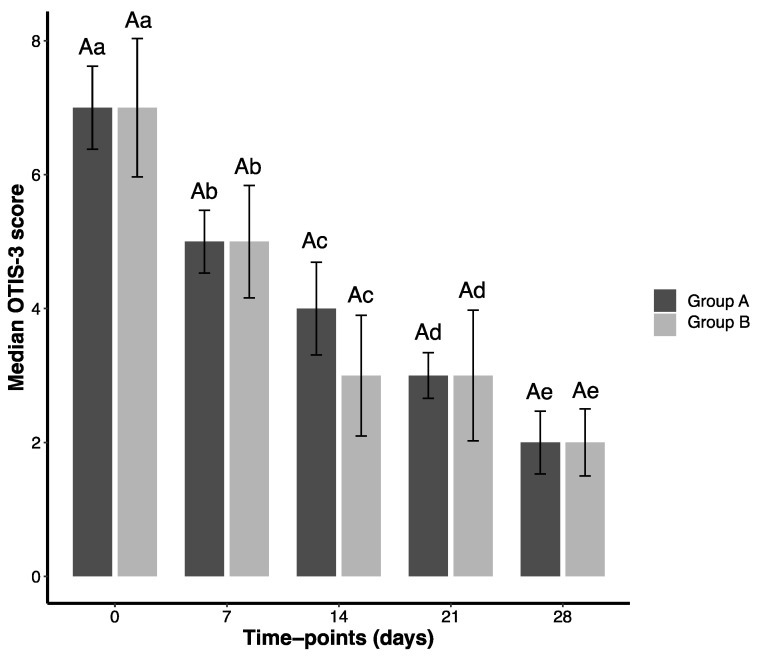
Median values (and standard error) obtained for the OTIS-3 assessment of 38 dogs presenting otitis externa and threatened with different ear solutions during 28 days and evaluated at five time points (T0, T7, T14, T21, T28). Group A (GA) comprised dogs threatened with a natural formula, meanwhile Group B (GB) comprised dogs treated with a conventional medication. Capital letters represent comparisons between groups (GA × GB). Lowercase letters represent comparisons within the same group. Sample size was the same at each time point (35 dogs in each group).

**Figure 3:**
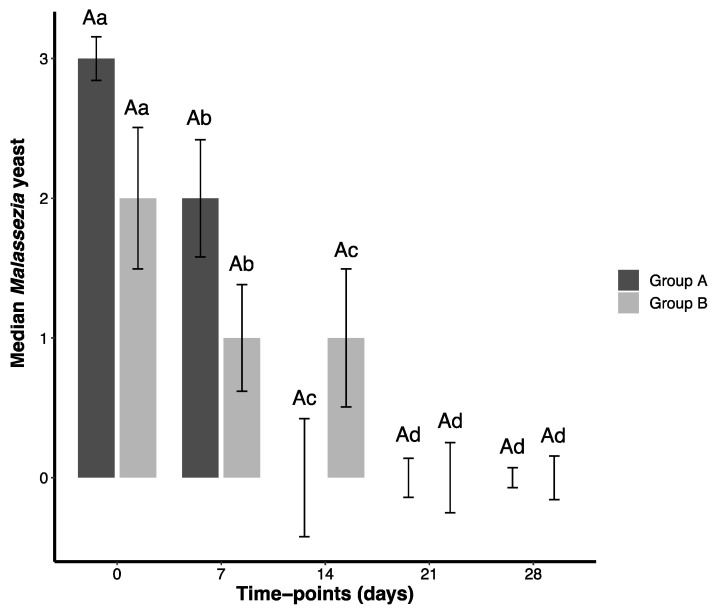
Median values (and standard error) obtained for the *Malassezia* yeast counting scores of 38 dogs presenting otitis externa and threatened with different ear solutions during 28 days and evaluated at five time points (T0, T7, T14, T21, T28). Group A (GA) comprised dogs threatened with a natural formula, meanwhile Group B (GB) comprised dogs treated with a conventional medication. Capital letters represent comparisons between groups (GA × GB). Lowercase letters represent comparisons within the same group. Sample size was the same at each time point (35 dogs in each group).

In summary, GA and GB showed comparable results for all parameters except bacilli counts. All Wilcoxon Signed Rank Test *p*-values are in Supplementary File S8. Bar plots for OTIS-3, pVAS, and *Malassezia* spp. are presented in Figure 1, Figure 2 and Figure 3. Coccoid and bacilli scores consistently yielded a median of 0.00, preventing satisfactory graphical representation (Supplementary Files S9 and S10).

### 3.5. Clinical Cases

Two clinical cases from each group (GA: C-01 and C-05; GB: C-06 and C-13) were selected, and their complete case information, assessment parameters and comparative photos from their ears and cytology exams are presented in Supplementary File S11.

## 4. Discussion

The prevalence of Lhasa Apso and Shih Tzu breeds in this study, aligning with their known predisposition to atopic dermatitis and subsequent otitis externa (OE) [27,28], is reflected by their popularity in Brazil and their genetic susceptibility to chronic dermatological conditions. Veterinary products formulated with safe, natural antimicrobials and anti-inflammatory agents may serve as effective first-line therapeutic options for managing dermatological conditions associated with microbial infections. In addition to their antimicrobial and anti-inflammatory properties, these compounds may also promote wound healing and overall skin health in dogs [29].

This study demonstrates the comparable efficacy of a novel topical formulation (Therapy A), containing antimicrobial peptides and plant extracts (chamomile, calendula, rosemary, hops), and a conventional treatment (Therapy B: gentamicin, clotrimazole, betamethasone) in managing canine OE. Both therapies yielded similar positive clinical outcomes across most assessed parameters: OTIS-3, pVAS, cytological evaluation, and pH. At T28, median OTIS-3 scores were 2 for both groups, with only 15.78% (3/19) of dogs in each group having scores above 4. Nuttal and Bensignor [21] define absence of OE at OTIS-3 < 4. Similarly, T28 median pVAS scores were 0 for GA and 1 for GB, with 21.05% (4/19) of dogs in each group still exhibiting mild itching (pVAS > 2) [22,23]. These findings reinforce the comparable efficacy of both treatments in managing otitis externa.

To our knowledge, this is the first study to combine plant extracts and antimicrobial peptides for canine OE, targeting a broad spectrum of microbial isolates. The efficacy of plant extracts against human and canine dermatological microbes is well-documented. Chamomile extract, for instance, has shown activity against *Staphylococcus aureus* and *P. aeruginosa* in human otitis [30,31]. A topical formulation with rosemary essential oil exhibited in vitro efficacy against *M. pachydermatis, Candida albicans*, and *P. aeruginosa*, and improved clinical and cytological outcomes in canine OE [32]. More recently, a lotion and shampoo containing lemon essential oil and the same antimicrobial peptides used in this study demonstrated comparable efficacy to conventional treatments (topical chlorhexidine + miconazole shampoo and oral cephalexin) for canine superficial pyoderma, notably achieving faster reductions in lesion and pruritus scores [33]. Another consideration is related to the composition of Therapy A, which combines antimicrobial peptides (bacterial-derived) with four different plant-derived extracts (chamomile, marigold, rosemary, and hops). These antimicrobial and anti-inflammatory combinations were a result of previous pre-clinical tests (results not shown), as specific ingredient suppliers were validated, which demonstrated a synergistic antimicrobial activity of the antimicrobial peptides and hop extract towards several strains of *Staphylococcus pseudintermedius* and *Malassezia pachydermatis*. This synergistic approach is derived from the different and complementary mechanisms in which the peptides and hop acids work against bacteria. The antimicrobial peptides are able to cause a rupture in the cytoplasmic membrane, causing leakage of essential metabolites and inhibiting several metabolic pathways. Moreover, they open a gate, where hop alpha and beta-acids can easily enter the microbial cell and acidify its interior, causing disruption of essential bacterial and fungal metabolism as well as intense oxidative stress. Moreover, the nanoencapsulated blend of chamomile, marigold and rosemary is a result of extensive in vitro screening on different extracts and their anti-inflammatory potential induced in macrophage cell culture by lipopolysaccharide (results not published). The specific blend used in Treatment A showed (3 μL/L of each plant extract) a similar effect to hydrocortisone acetate at 9 μM in TNF-α inhibition. These extracts contain apigenin, bisabolol, flavonoids and rosmarinic acid, which are able to inhibit several inflammatory pathways, such as the production of prostaglandins and leukotrienes by inhibiting the enzymes COX-2 and LOX, respectively [29,34]. Therefore, the combination of these ingredients led to the development of Treatment A, taking into consideration that otitis externa is largely rooted in microbial infection and inflammation.

No significant differences in pinnal pH were observed between or within groups throughout the study. While alterations in cutaneous pH are associated with skin disorders and disease predisposition [35], and lower pH values are typically found in healthy human ear canals compared to those with OE [36], our findings did not correlate pH changes with treatment improvements in canine OE. This contrasts with observations in humans, where repeated exposure to alkalinizing agents can impair skin barrier function, particularly in atopic individuals [37].

Regarding safety, only one dog in GB experienced an adverse drug reaction (erythema and swelling), leading to its removal from the study. In contrast, no cutaneous drug reaction signs were observed in any dog treated with Therapy A, suggesting a favorable safety profile for the novel formulation. A previous study has also shown that a topical medication based on gentamicin, clotrimazole, betamethasone, and benzocaine was associated with a delayed hypersensitivity reaction leading to erythema, pain, and ear discharge in a dog [38].

The prevalence of staphylococci (38.3% of total isolates) in our canine OE cases contrasts with a previous study conducted in Brazil, which reported 60% staphylococcal involvement [39]. This discrepancy may stem from methodological differences. Our use of non-selective media and MALDI-TOF identification of various colony morphologies likely allowed for broader bacterial diversity, including potentially saprophytic or transient species [40]. Conversely, the previous study used selective mannitol salt agar, which may have favored staphylococcal detection.

*Staphylococcus coagulans*, a coagulase-positive species, was the most prevalent bacterial species (16.7%) in our study, consistent with previous reports in canine OE [39,41,42,43,44]. This species, recently reclassified from *Staphylococcus schleiferi* subsp. *coagulans* [45], has shown distinct host adaptation patterns compared to *S. schleiferi* [42]. While *S. schleiferi* is often associated with human infections and tends to exhibit greater oxacillin resistance [46,47,48], our study observed higher oxacillin resistance rates in *S. coagulans*. Nevertheless, *S. schleiferi* was the fifth most prevalent species, and its public health implications, including potential zoonotic transmission to immunocompetent humans [49], denote an important issue that should be continually addressed by the scientific community.

The high prevalence of *Proteus mirabilis* (15% of all isolates), particularly in GA, is notable. *Proteus* spp. are typically linked to chronic or complicated OE, and factors like ear canal humidity, compromised skin barrier, or prior antiseptic use may contribute to their presence. Our non-selective culture methods may have also enhanced Gram-negative organism detection. Coagulase-positive staphylococci, including *S. pseudintermedius* (the third most common species in this study), are frequently isolated from OE cases, with reports exceeding 70% [50]. *Staphylococcus pseudintermedius*, a commensal of canine skin and mucous membranes [51], is a primary contributor to ear dysbiosis in atopic dogs [52]. The combined prevalence of *S. coagulans* and *S. pseudintermedius* in our study (34.21% of dogs with OE) suggests a diverse OE etiology within the studied population. Moreover, although overall clinical outcomes were comparable between the two groups, bacilli count significantly decreased in the conventional therapy group (GB) only, whereas no significant reduction was observed in the novel therapy group (GA). This suggests that the conventional formulation may have greater efficacy against bacillary infections, which are often considered more difficult to manage in clinical practice. It is also important to state that bacilli infections were seldom found in the present study.

MALDI-TOF analysis also identified a few rare or occasional isolates. While these microorganisms were detected in very low numbers and may represent contaminants introduced during sample collection or handling, it is also possible that they reflect part of the complex microbial community of the canine ear canal. Their clinical relevance in the pathogenesis of otitis externa remains uncertain, particularly in cases where they were not consistently associated with cytological or clinical evidence of infection. This interpretation is consistent with previous studies describing the complexity of the canine ear microbiota and the occurrence of less common taxa in affected and healthy dogs [5,7].

The global concern of antimicrobial resistance (AMR) was highlighted by the identification of three multidrug-resistant (MDR) isolates (C-01, C-05, C-09), all from GA. Despite these AMR strains, all three dogs achieved pVAS scores of 0 by the end of the treatment. While only one dog with MDR isolates (C-09) had an OTIS-3 score above 4 at T28, it showed significant improvement from baseline (9–11 at T0 to 6 at T28), indicating potential for further improvement with prolonged treatment. Cytologically, C-05 was the only dog to maintain a score of 2, while the other two achieved scores of 0. This indicates that Therapy A was clinically effective in resolving OE in two out of three cases involving MDR bacteria. Methicillin (oxacillin)-resistant *S. aureus* (MRSA) is a major pathogen in various environments [52]. Although *S. aureus* was not identified, 18.75% (3/16) of tested staphylococcal isolates were methicillin-resistant, a higher prevalence than previously reported rates (3–11%) in canine OE [39,53,54,55], raising concerns about increasing AMR in these cases.

Limitations of the present study should be discussed. While the open and multicentric design provides a broader diversity of data, it may present challenges in obtaining rigorous control, as the administration of the tested formulations was entrusted to the dog owners, potentially leading to therapeutic failures. The open-label design, although justified by the distinct sensory and physical characteristics of the tested formulations, remains a methodological limitation, as the absence of blinding could have introduced observer or owner bias. Nevertheless, this potential bias was mitigated by ensuring that all evaluations were performed by the same trained clinician using standardized scoring systems. Ideally, the treatment of all dogs by the same individual would have been preferable. However, since the selected dogs were sourced from the dermatology services of veterinary clinics and were not experimental animals, this approach was impractical.

Additionally, the sample size was determined by convenience based on the number of eligible cases during the study period, and no prior statistical power calculation was performed. This limitation may affect the study’s statistical power and the generalizability of its findings to broader canine populations. Furthermore, no correction for multiple comparisons (such as Bonferroni adjustment) was applied to the statistical analyses. Given the number of time points and outcome variables evaluated, this may have increased the likelihood of Type I error and should be acknowledged as a statistical limitation.

Additionally, in an ideal scenario, all dogs should have the same underlying disease and receive identical treatment. This condition was impracticable due to the varying severity of the same disease in different animals, differences in response to the same treatment, and the financial constraints of their owners. The underlying causes of otitis externa were heterogeneous and not stratified, making it impossible to determine whether therapy A maintains consistent efficacy across different primary etiologies, such as allergic skin disease, anatomical predisposition, or microbial dysbiosis. Future studies should stratify patients according to primary cause to better assess the reproducibility of these findings. Additionally, the treatment period was limited to four weeks, and no long-term follow-up was performed. Extended monitoring will be important in future trials to determine relapse rates and long-term clinical outcomes.

Finally, detailed characterization of the antimicrobial peptides from therapy A was not allowed due to confidentiality restrictions, which limits the reproducibility and mechanistic understanding of current findings. Despite these limitations, our findings strongly suggest that the tested therapy has great potential as an alternative and safer treatment for canine otitis externa.

## 5. Conclusions

This study demonstrates that a novel topical formulation, combining antimicrobial peptides and encapsulated plant extracts (chamomile, calendula, rosemary, hops), offers comparable efficacy to conventional OE treatment (gentamicin, clotrimazole, betamethasone) in dogs. Both approaches significantly reduced inflammation, pruritus, and microbial presence.

Notably, the alternative therapy effectively resolved OE even against multi-resistant bacterial strains, addressing a key concern in veterinary antimicrobial resistance (AMR). Furthermore, its favorable safety profile was evidenced by the absence of adverse drug reactions. These findings strongly support integrating plant-derived bioactives and antimicrobial peptides into dermatological treatments as effective, antibiotic-free, and corticoid-sparing alternatives for canine OE, potentially shifting current management paradigms. Future research should explore long-term disease control, microbiome modulation, and expanded clinical applications.

## Figures and Tables

**Table 1:** Information regarding ID, group assignment, breed, sex, age, sterilization status and number of ears affected of each dog included in the present study.

ID	Group	Breed	Sex	Age	Surgically Sterilized	Ear Affected
C-01	A	Lhasa Apso	Male	14	Yes	Both
C-02	A	Lhasa Apso	Female	16	Yes	Right
C-03	A	Lhasa Apso	Female	7	Yes	Both
C-04	B	Beagle	Male	7	Yes	Left
C-05	A	German Shephard	Female	7	No	Both
C-06	B	Shih Tzu	Male	8	Yes	Both
C-07	B	Lhasa Apso	Female	6	Yes	Left
C-08	B	Shih Tzu	Male	7	Yes	Both
C-09	A	Golden Retriever	Female	3	Yes	Both
C-10	B	Golden Retriever	Male	1	Yes	Both
C-11	A	Pit Bull	Female	3	No	Both
C-12	B	Mixed	Male	12	Yes	Both
C-13	B	Shar-Pei	Female	2	Yes	Both
C-14	B	Shih Tzu	Female	4	Yes	Both
C-15	B	Lhasa Apso	Male	10	Yes	Both
C-17	A	Mixed	Female	3	Yes	Left
C-18	A	Mixed	Male	3	Yes	Both
C-19	A	Schnauzer	Female	5	Yes	Right
C-20	B	Golden Retriever	Female	4	Yes	Right
C-21	A	Dachshund	Female	10	Yes	Both
C-22	B	Shih Tzu	Male	10	Yes	Both
PG-01	A	Shih Tzu	Female	5	Yes	Both
PG-02	A	Lhasa Apso	Female	4	Yes	Both
PG-03	B	Poodle	Female	7	Yes	Both
PG-04	A	Lhasa Apso	Male	8	Yes	Both
PG-05	A	Mixed	Male	6	Yes	Both
PG-06	A	Lhasa Apso	Male	1	Yes	Both
PG-07	B	Mixed	Female	6	Yes	Both
PG-09	A	French bulldog	Male	2	Yes	Both
PG-09	B	Chihuahua	Female	3	Yes	Both
PG-10	A	Mixed	Female	3	Yes	Both
PG-11	B	Lhasa	Male	8	Yes	Both
PG-12	B	Male	Male	9	Yes	Both
PG-13	B	Labrador	Male	3	Yes	Both
PG-14	B	Mixed	Male	4	Yes	Both
PG-15	A	Lhasa Apso	Male	2	Yes	Both
PG-16	B	Mixed	Female	8	Yes	Both
PG-17	A	Mixed	Male	5	Yes	Both

C = dogs sampled in Curitiba. PG = dogs sampled in Ponta Grossa.

**Table 2:** Species distribution of 60 microorganisms isolated from the ear canal of dogs presenting OE. Identification was performed using microbial protein fingerprint by MALDI-TOF.

Microorganism	Number of Isolates	Proportion (%)
*Bacillus cereus*	3	5.0
*Bacillus megaterium*	2	3.33
*Bacillus pumillus*	2	3.33
*Enterococcus canintestini*	1	1.67
*Enterococcus faecalis*	1	1.67
*Escherichia coli*	2	3.33
*Klebsiella planticola*	1	1.67
*Klebsiella variicola*	1	1.67
*Leclercia adecarboxylata*	1	1.67
*Malassezia pachydermatis*	3	5.0
*Paenibacillus lautus*	1	1.67
*Paenibacillus nematophilus*	1	1.67
*Proteus mirabilis*	9	15.0
*Pseudomonas aeruginosa*	4	6.67
*Pseudomonas koreensis*	1	1.67
*Psychrobacter lutiphocae*	1	1.67
*Raoultella* (*K.*) *ornithinolytica*	1	1.67
*Staphylococcus coagulans*	10	16.7
*Staphylococcus intermedius*	1	1.67
*Staphylococcus pseudintermedius*	6	10.0
*Staphylococcus schleiferi*	4	6.67
*Staphylococcus saprophyticus* subsp. *saprophyticus*	1	1.67
*Staphylococcus warneri*	1	1.67
*Weissella cibaria*	1	1.67
*Weissella confusa*	1	1.67
Total	60	100.0

## Data Availability

Full data is available upon request.

## Supplementary Materials

The following supporting information can be downloaded at https://www.mdpi.com/article/10.3390/pathogens14111112/s1, Supplementary File S1: Antibiotics tested for each identified bacterial group. An X in the table indicates that the respective bacterial group was tested with the corresponding antibiotic; Supplementary File S2: Clinical, microbiological, and pH evaluation of dogs treated for otitis externa. Median values for OTIS-3, pVAS, and cytological scores (Malassezia spp., cocci, and bacilli), along with mean pH values, are presented for dogs treated with either a novel ear solution containing natural antimicrobials (GA) or a conventional formulation containing gentamicin, betamethasone valerate, and micronized clotrimazole (GB). Dogs were assessed weekly over 28 days (T0 to T28); Supplementary File S3: Individual results of each dog for all parameters analyzed; Supplementary File S4: Microorganisms identified in dogs presenting OE in the present study. Automatized identification was performed using MALDI-TOF system; Supplementary File S5: Antibiogram results for each isolate; Supplementary File S6: Prevalence of resistant isolates according to the tested antibiotics; Supplementary File S7: Statistical comparison between treatments A and B for all parameters analyzed; Supplementary File S8: Statistical comparison within treatments A and B throughout the tested period for all parameters analyzed; Supplementary File S9: Graphical result on bacilli cytology; Supplementary File S10: Graphical result on cocci cytology; Supplementary File S11: Case studies.

## Abbreviations

The following abbreviations are used in this manuscript:

OE	Otitis externa
AMR	Antimicrobial resistance
AD	Atopic dermatitis
AMP(s)	Antimicrobial peptide(s)
OTIS-3	Otitis Index Scoring System
pVAS	Pruritus Visual Analog Scale
PUCPR	Pontifícia Universidade Católica do Paraná
GA	Group A (Therapy A: antimicrobial peptides + plant extracts)
GB	Group B (Therapy B: gentamicin + betamethasone valerate + clotrimazole)
MSD	Merck Sharp & Dohme Animal Health
MALDI-TOF MS	Matrix-Assisted Laser Desorption/Ionization–Time of Flight Mass Spectrometry
CLSI	Clinical and Laboratory Standards Institute
CNPq	Conselho Nacional de Desenvolvimento Científico e Tecnológico (Brazilian National Council for Scientific and Technological Development)
MDR	Multidrug resistant
