# Supplementary material for: Comparative Efficacy of a Novel Topical Formulation with Antimicrobial Peptides and Encapsulated Plant Extracts Versus Conventional Therapies for Canine Otitis Externa
Source: Pathogens. 2025 Nov 1;14(11):1112. doi: 10.3390/pathogens14111112 (PMC12655140; doi:10.3390/pathogens14111112)
Supplement: Supplementary file 1 [file pathogens-14-01112-s001.zip › Supplementary File S1.pdf]

Supplementary File 1. Antibiotics tested for each identified bacterial group. An X in the table indicates that the respective bacterial group was tested with the corresponding antibiotic.

Class	Antibiotic	***Pseudomonas* spp.**	**Enterobacteriaceae**	***Enterococcus* spp.**	***Staphylococcus* spp. (except *S. warneri*)**
Aminoglycosides	Amikacin (AMI)	X	X		
	Gentamicin (GEN)	X	X		X
Carbapenems	Meropenem (MPM)	X	X		
Cephalosporins	Ceftriaxone (CEF)		X		
	Cephalexin (CFE)				X
	Cefepime (CPM)	X			
Glycopeptides	Vancomycin (VAN)			X	
Lincosamides	Clindamycin (CLI)				X
Macrolides	Azithromycin (AZI)			X	X
	Erithromycin (ERI)			X	
Penicillins	Ampicillin (AMP)		X	X	
	Piperacillin + Tazobactam (TZP)	X			
	Oxacillin (OXA)				X
Tetracyclines	Doxycycline (DOX)			X	X
Penicillin-like	Amoxicillin + Clavulanate (AMC)		X	X	
