# Supplementary material for: Comparative Efficacy of a Novel Topical Formulation with Antimicrobial Peptides and Encapsulated Plant Extracts Versus Conventional Therapies for Canine Otitis Externa
Source: Pathogens. 2025 Nov 1;14(11):1112. doi: 10.3390/pathogens14111112 (PMC12655140; doi:10.3390/pathogens14111112)
Supplement: Supplementary file 1 [file pathogens-14-01112-s001.zip › Supplementary File S10.pdf]

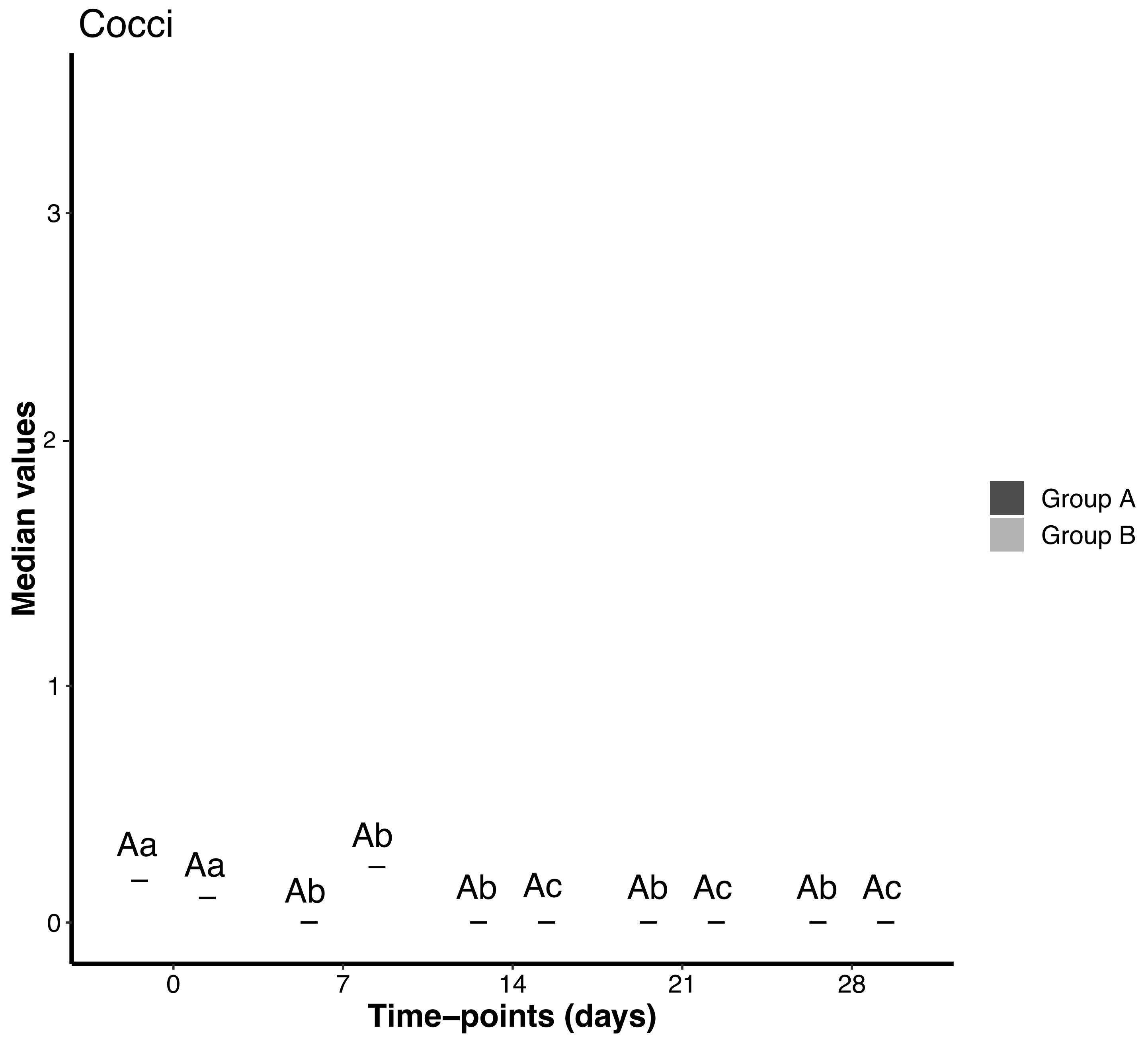
Cocci
Median values
3
2
1
0
Aa
Aa
Ab
Ab
Ab
Ac
Ab
Ac
Ab
Ac
0
7
14
21
28
Time−points (days)
Group A
Group B
