# Supplementary material for: Comparative Efficacy of a Novel Topical Formulation with Antimicrobial Peptides and Encapsulated Plant Extracts Versus Conventional Therapies for Canine Otitis Externa
Source: Pathogens. 2025 Nov 1;14(11):1112. doi: 10.3390/pathogens14111112 (PMC12655140; doi:10.3390/pathogens14111112)
Supplement: Supplementary file 1 [file pathogens-14-01112-s001.zip › Supplementary File S11.pdf]

Supplementary File 11

<u>Case 1</u>

Dog: C-01
Group: A
Breed: Lhasa Apso
Sex: Male
Age: 14 years-old
Sterilized: yes
Number of ears affected: Both
Environment: Lives in an apartment without contact with other dogs or pets
Behavior: Generally friendly, allows handling when muzzled

Microbial species isolated in T0:
Left: *Staphylococcus warneri*
Right: N/I

Table 2: Assessment of parameters from dog C-01.

	**T0**	**T7**	**T14**	**T21**	**T28**
pVAS (left)	0	0	0	0	0
pVAS (right)	4	3	2	1	0
OTIS-3 (left)	3	3	3	4	2
OTIS-3 (right)	6	4	4	4	2
Malassezia (left)	3	0	0	0	0
Malassezia (right)	2	2	0	0	0
Cocci (left)	0	0	0	0	0
Cocci (right)	0	0	0	0	0
Bacilli (left)	0	0	0	0	0
Bacilli (right)	0	0	0	0	0
pH (left)	6.32	6.84	6.8	6.54	7.25
pH (right)	5.38	5.76	5.92	6.3	7.27

C-01

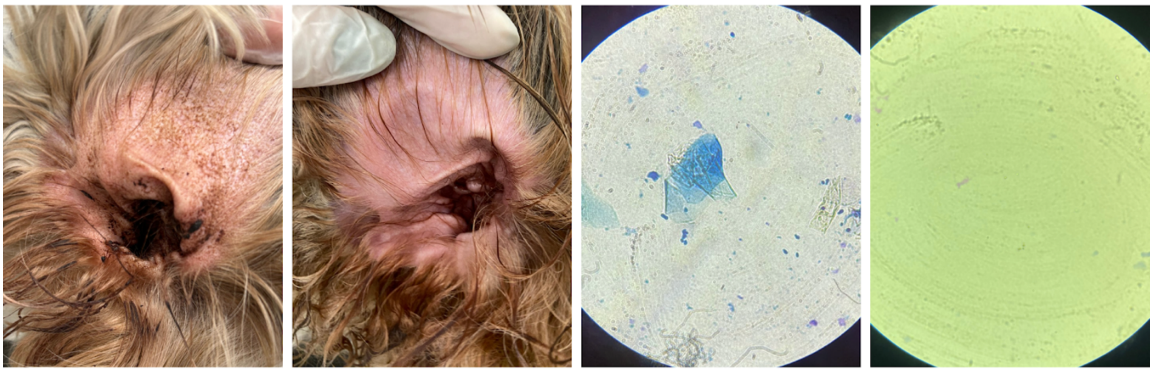

T0 T28 T0 T28

Figure 2: Comparative of ear aspect and cytology of T0 and T28 from dog C-01 (right ear).

<u>Case 2</u>

Dog: C-18
Group: A
Breed: mixed breed
Sex: male
Age: 3
Sterilized: yes
Number of ears affected: Both
Environment: Lives in a house with contact with other dogs or pets
Behavior: Very friendly, easy handling

Microbial species isolated in T0:
Left: *Bacillus pumillus*
Right: *Bacillus pumillus*

Table 1: Assessment of parameters from dog C-05.

	**T0**	**T7**	**T14**	**T21**	**T28**
pVAS (left)	3	2.5	2	0	0
pVAS (right)	3	2.5	2	0	0
OTIS-3 (left)	6	5	4	3	3
OTIS-3 (right)	6	5	5	3	3
Malassezia (left)	3	3	1	0	0
Malassezia (right)	3	3	3	0	0
Cocci (left)	1	0	0	0	0
Cocci (right)	1	0	0	0	0
Bacilli (left)	1	0	0	0	0
Bacilli (right)	1	0	0	0	0
pH (left)	5.94	6.16	5.85	6.74	7.01
pH (right)	5.62	5.97	5.67	6.52	7.07

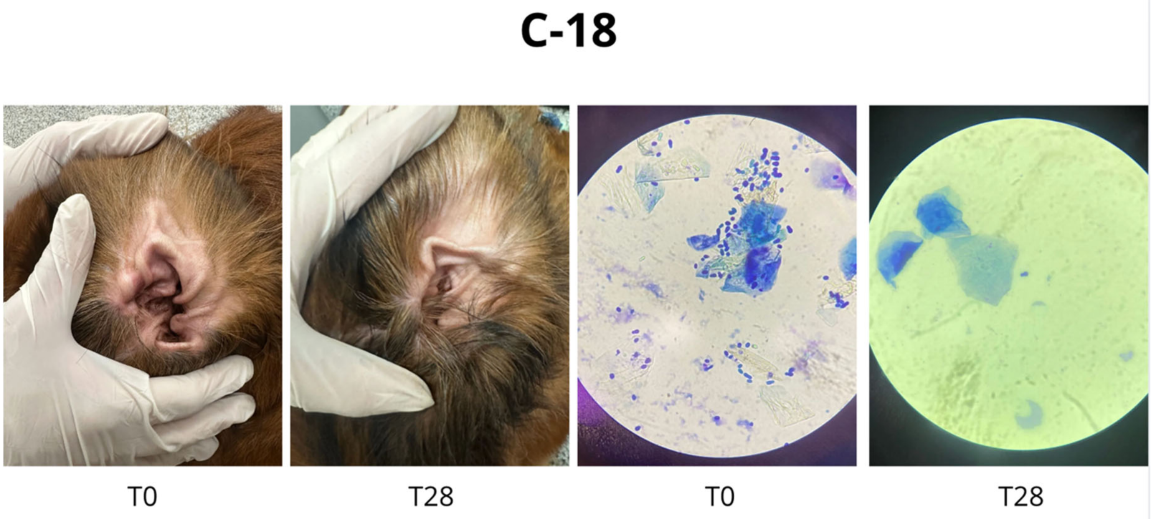

Figure 1: Comparative of ear aspect and cytology of T0 and T28 from dog C-05 (left ear).

<u>Case 3</u>

Dog: C-06
Group: B
Breed: Lhasa Apso
Sex: Male
Age: 8 years-old

Sterilized: Yes
Number of ears affected: both
Environment: Lives in an apartment without contact with other dogs or pets
Behavior: Very friendly, easy handling

Microbial species isolated in T0:
Left: *Staphylococcus schleiferi* subsp. *coagulans*
Right: *Staphylococcus schleiferi* subsp. *schleiferi*

Table 3: Assessment of parameters from dog C-06.

	**T0**	**T7**	**T14**	**T21**	**T28**
pVAS (left)	9	5	3	1	0
pVAS (right)	9	5	3	1	0
OTIS-3 (left)	3	1	0	2	2
OTIS-3 (right)	8	4	2	2	2
Malassezia (left)	2	1	0	0	0
Malassezia (right)	2	0	0	0	0
Cocci (left)	0	0	0	0	0
Cocci (right)	0	0	0	0	0
Bacilli (left)	0	0	0	0	0
Bacilli (right)	0	0	0	0	0
pH (left)	5.66	5.80	5.24	5.76	5.93
pH (right)	5.72	5.92	5.16	5.58	6.87

C-06

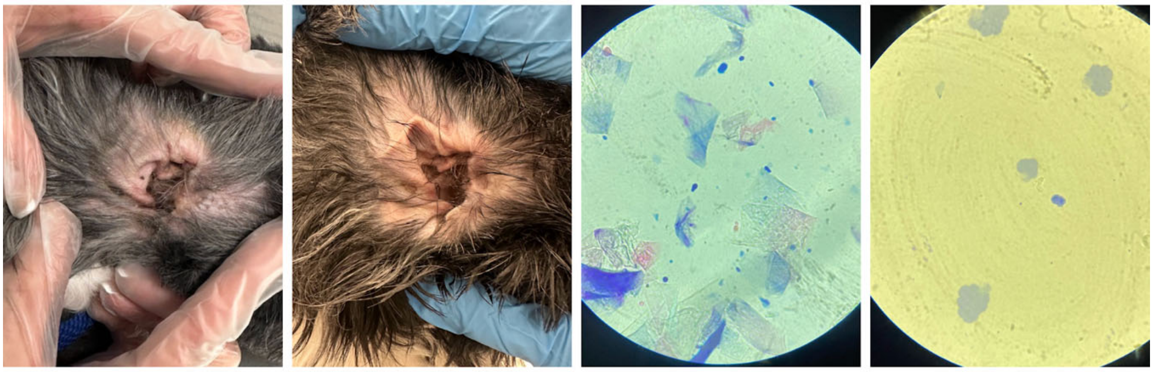

Figure 3: Comparative of ear aspect and cytology of T0 and T28 from dog C-06 (right ear).

<u>Case 4</u>

Dog: C-13
Group: B
Breed: Shar-Pei
Sex: Female
Age: 2 years-old
Sterilized: Yes
Number of ears affected: Both
Environment: Lives in a house, with sporadic contact with other dogs
Behavior: Very friendly, easy handling

Microbial species isolated in T0:
Left: *Staphylococcus schleiferi* subsp. *coagulans* and *Enterococcus canintestini*
Right: N/I

Table 3: Assessment of parameters from dog C-13.

	**T0**	**T7**	**T14**	**T21**	**T28**
pVAS (left)	7	5	5	3	1
pVAS (right)	7	5	5	3	1
OTIS-3 (left)	7	7	6	4	3
OTIS-3 (right)	8	7	6	4	3
Malassezia (left)	3	3	2	0	0
Malassezia (right)	3	3	0	0	0
Cocci (left)	3	3	0	0	0
Cocci (right)	3	0	0	0	0
Bacilli (left)	0	0	0	0	0
Bacilli (right)	0	0	0	0	0
pH (left)	5.81	5.67	6.19	6.10	6.18
pH (right)	5.28	5.7	5.9	5.85	8.06

C-13

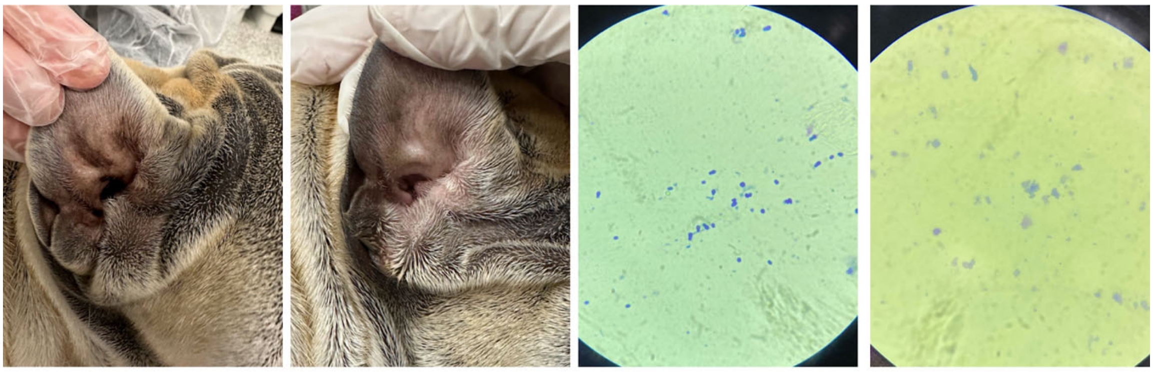

T0 T28 T0 T28

Figure 4: Comparative of ear aspect and cytology of T0 and T28 from dog C-013 (right ear).
