# Supplementary material for: Comparative Efficacy of a Novel Topical Formulation with Antimicrobial Peptides and Encapsulated Plant Extracts Versus Conventional Therapies for Canine Otitis Externa
Source: Pathogens. 2025 Nov 1;14(11):1112. doi: 10.3390/pathogens14111112 (PMC12655140; doi:10.3390/pathogens14111112)
Supplement: Supplementary file 1 [file pathogens-14-01112-s001.zip › Supplementary File S2.pdf]

**Supplementary File 2.** Clinical, microbiological, and pH evaluation of dogs treated for otitis externa. Median values for OTIS-3, pVAS, and cytological scores (*Malassezia* spp., cocci, and bacilli), along with mean pH values, are presented for dogs treated with either a novel ear solution containing natural antimicrobials (GA) or a conventional formulation containing gentamicin, betamethasone valerate, and micronized clotrimazole (GB). Dogs were assessed weekly over 28 days (T0 to T28).

| Group | Assessment | Value | T0 | T7 | T14 | T21 | T28 |
|---|---|---|---|---|---|---|---|
| **A** | OTIS-3 | Median | 7.00 | 5.00 | 4.00 | 3.00 | 2.00 |
| | | (SE) | (0.62) | (0.46) | (0.69) | (0.34) | (0.46) |
| | pVAS | Median | 8.00 | 4.50 | 3.00 | 2.00 | 0.00 |
| | | (SE) | (0.71) | (0.82) | (1.09) | (0.99) | (0.39) |
| | pH | Mean | 6.06 | 5.80 | 6.03 | 6.15 | 6.08 |
| | | (SD) | (0.58) | (0.46) | (0.77) | (0.86) | (0.99) |
| | *Malassezia* spp. | Median | 3.00 | 2.00 | 0.00 | 0.00 | 0.00 |
| | | (SE) | (0.15) | (0.41) | (0.42) | (0.14) | (0.07) |
| | Cocci | Median | 0.00 | 0.00 | 0.00 | 0.00 | 0.00 |
| | | (SE) | (0.17) | (0.0) | (0.0) | (0.0) | (0.0) |
| | Bacilli | Median | 0.00 | 0.00 | 0.00 | 0.00 | 0.00 |
| | | (SE) | (0.0) | (0.0) | (0.0) | (0.0) | (0.0) |
| **B** | OTIS-3 | Median | 7.00 | 5.00 | 3.00 | 3.00 | 2.00 |
| | | (SE) | (1.03) | (0.84) | (0.90) | (0.97) | (0.50) |
| | pVAS | Median | 7.00 | 5.00 | 4.00 | 1.00 | 1.00 |
| | | (SE) | (0.47) | (0.38) | (0.83) | (0.77) | (0.55) |
| | pH | Mean | 6.10 | 5.86 | 5.99 | 6.04 | 6.30 |
| | | (SD) | (0.8) | (0.5) | (0.97) | (1.02) | (1.09) |
| | *Malassezia* spp. | Median | 2.00 | 1.00 | 1.00 | 0.00 | 0.00 |
| | | (SE) | (0.50) | (0.38) | (0.49) | (0.25) | (0.15) |
| | Cocci | Median | 0.00 | 0.00 | 0.00 | 0.00 | 0.00 |
| | | (SE) | (1.03) | (0.23) | (0.0) | (0.0) | (0.0) |
| | Bacilli | Median | 0.00 | 0.00 | 0.00 | 0.00 | 0.00 |
| | | (SE) | (0.31) | (0.03) | (0.0) | (0.0) | (0.0) |

SE = standard error; SD = standard deviation.
