# Supplementary material for: Comparative Efficacy of a Novel Topical Formulation with Antimicrobial Peptides and Encapsulated Plant Extracts Versus Conventional Therapies for Canine Otitis Externa
Source: Pathogens. 2025 Nov 1;14(11):1112. doi: 10.3390/pathogens14111112 (PMC12655140; doi:10.3390/pathogens14111112)
Supplement: Supplementary file 1 [file pathogens-14-01112-s001.zip › Supplementary File S4.pdf]

Supplementary File 4. Microorganisms identified in dogs presenting OE in the present study. Automatized identification was performed using MALDI-TOF system.

ID	Ear	Group	Identified microorganism
C-01	Left	A	*Staphylococcus warneri*
C-01	Right	A	N/I
C-02	Left	A	N/I
C-03	Left	A	*Staphylococcus coagulans*
C-03	Right	A	*Staphylococcus coagulans*
C-04	Left	B	*Pseudomonas aeruginosa*
C-05	Left	A	*Proteus mirabilis*
C-05	Right	A	*Staphylococcus pseudintermedius*
C-05	Right	A	*Pseudomonas aeruginosa*
C-06	Left	B	*Staphylococcus coagulans*
C-06	Right	B	*Staphylococcus schleiferi*
C-07	Left	B	*Staphylococcus pseudintermedius*
C-08	Left	B	*Staphylococcus coagulans*
C-08	Right	B	*Staphylococcus coagulans*
C-09	Left	A	*Staphylococcus pseudintermedius*
C-09	Right	A	*Pseudomonas aeruginosa*
C-10	Left	B	N/I
C-10	Right	B	N/I
C-11	Left	A	*Staphylococcus schleiferi*
C-11	Right	A	*Proteus mirabilis*
C-12	Left	B	N/I
C-12	Right	B	N/I
C-13	Left	B	*Staphylococcus coagulans*
C-13	Left	B	*Enterococcus canintestini*
C-13	Right	B	N/I
C-14	Left	B	*Staphylococcus coagulans*
C-14	Right	B	*Escherichia coli*
C-15	Left	B	*Staphylococcus coagulans*
C-15	Right	B	*Staphylococcus coagulans*
C-17	Left	A	N/I
C-18	Left	A	*Bacillus pumillus*
C-18	Right	A	*Bacillus pumillus*
C-19	Right	A	*Staphylococcus pseudintermedius*
C-20	Right	B	*Weissella confusa*
C-21	Left	A	*Malassezia pachydermatis*
C-21	Right	A	N/I
C-22	Left	B	*Staphylococcus coagulans*
C-22	Left	B	*Staphylococcus saprophyticus* subsp. *saprophyticus*
C-22	Right	B	N/I
PG-01	Left	A	*Klebsiella planticola*
PG-01	Left	A	*Proteus mirabilis*
PG-01	Left	A	*Raoultella (K.) ornithinolytica*

PG-02	Left	A	*Staphylococcus schleiferi*
PG-02	Left	A	*Proteus mirabilis*
PG-02	Right	A	*Staphylococcus schleiferi*
PG-02	Right	A	*Proteus mirabilis*
PG-03	Left	B	*Pseudomonas koreensis*
PG-03	Left	B	*Klebsiella variicola*
PG-03	Right	B	*Staphylococcus intermedius*
PG-03	Rigt	B	*Malassezia pachydermatis*
PG-04	Left	A	*Enterococcus faecalis*
PG-04	Left	A	*Proteus mirabilis*
PG-04	Left	A	*Escherichia coli*
PG-04	Right	A	*Psychrobacter lutiphocae*
PG-05	Left	A	*Bacillus cereus*
PG-05	Right	A	N/I
PG-06	Left	A	*Proteus mirabilis*
PG-06	Right	A	N/I
PG-07	Left	B	N/I
PG-07	Right	B	N/I
PG-08	Left	A	*Proteus mirabilis*
PG-08	Left	A	*Proteus mirabilis*
PG-08	Left	A	*Staphylococcus pseudintermedius*
PG-09	Left	B	*Paenibacillus nematophilus*
PG-09	Right	B	*Leclercia adecarboxylata*
PG-09	Right	B	*Bacillus cereus*
PG-10	Left	A	*Bacillus cereus*
PG-10	Left	A	*Staphylococcus pseudintermedius*
PG-10	Right	A	*Malassezia pachydermatis*
PG-11	Left	B	N/I
PG-11	Right	B	N/I
PG-12	Left	B	N/I
PG-12	Right	B	N/I
PG-13	Left	B	*Pseudomonas aeruginosa*
PG-13	Right	B	*Bacillus megaterium*
PG-14	Left	B	N/I
PG-14	Right	B	*Bacillus megaterium*
PG-15	Left	A	N/I
PG-15	Right	A	N/I
PG-16	Left	B	*Paenibacillus lautus*
PG-16	Right	B	N/I
PG-17	Left	A	*Weissella cibaria*
PG-17	Right	A	N/I

N/I = absence of colonies in microbiological culture.
