# Supplementary material for: Comparative Efficacy of a Novel Topical Formulation with Antimicrobial Peptides and Encapsulated Plant Extracts Versus Conventional Therapies for Canine Otitis Externa
Source: Pathogens. 2025 Nov 1;14(11):1112. doi: 10.3390/pathogens14111112 (PMC12655140; doi:10.3390/pathogens14111112)
Supplement: Supplementary file 1 [file pathogens-14-01112-s001.zip › Supplementary File S6.pdf]

Supplementary File 6. Prevalence of resistant isolates according to the tested antibiotics

Bacteria	Number of isolates	AMI	GEN	MPM	CEF	CPM	VAN	CLI	AZI	ERI	AMP	TZP	OXA	DOX	AMC
Enterococcus canintestini	1						0%		0%	0%	0%				0%
Escherichia coli	1	0%	0%	100%	0%						100%				0%
Pseudomonas aeruginosa	3	0%	0%	0%		33,33%						0%			
Proteus mirabilis	2	0%	0%	50%	0%						50%				0%
Staphylococcus coagulans	10		0%		20%			10%	30%				20%	0%	
Staphylococcus pseudintermedius	4		0%		25%			75%	50%				25%	75%	
Staphylococcus schleiferi	2		0%		0%			0%	0%				0%	0%	
Staphylococcus warneri	1		100%					100%	100%					0%	

AMI = amikacin; GEN = gentamicin; MPM = meropenem; CEF = ceftriaxone; CFE = cephalexin; CPM = cefepime; VAN = vancomycin; CLI = clindamycin; AZI = azithromycin; ERI = erythromycin; AMP = ampicillin; TZP = piperacillin + tazobactam; OXA = oxacillin; DOX = doxycycline; AMC = amoxicillin + clavulanate; Absent values = not tested.
