# Supplementary figures and images for: Comparative Efficacy of a Novel Topical Formulation with Antimicrobial Peptides and Encapsulated Plant Extracts Versus Conventional Therapies for Canine Otitis Externa
Source: Pathogens. 2025 Nov 1;14(11):1112. doi: 10.3390/pathogens14111112 (PMC12655140; doi:10.3390/pathogens14111112)

# Bacilli

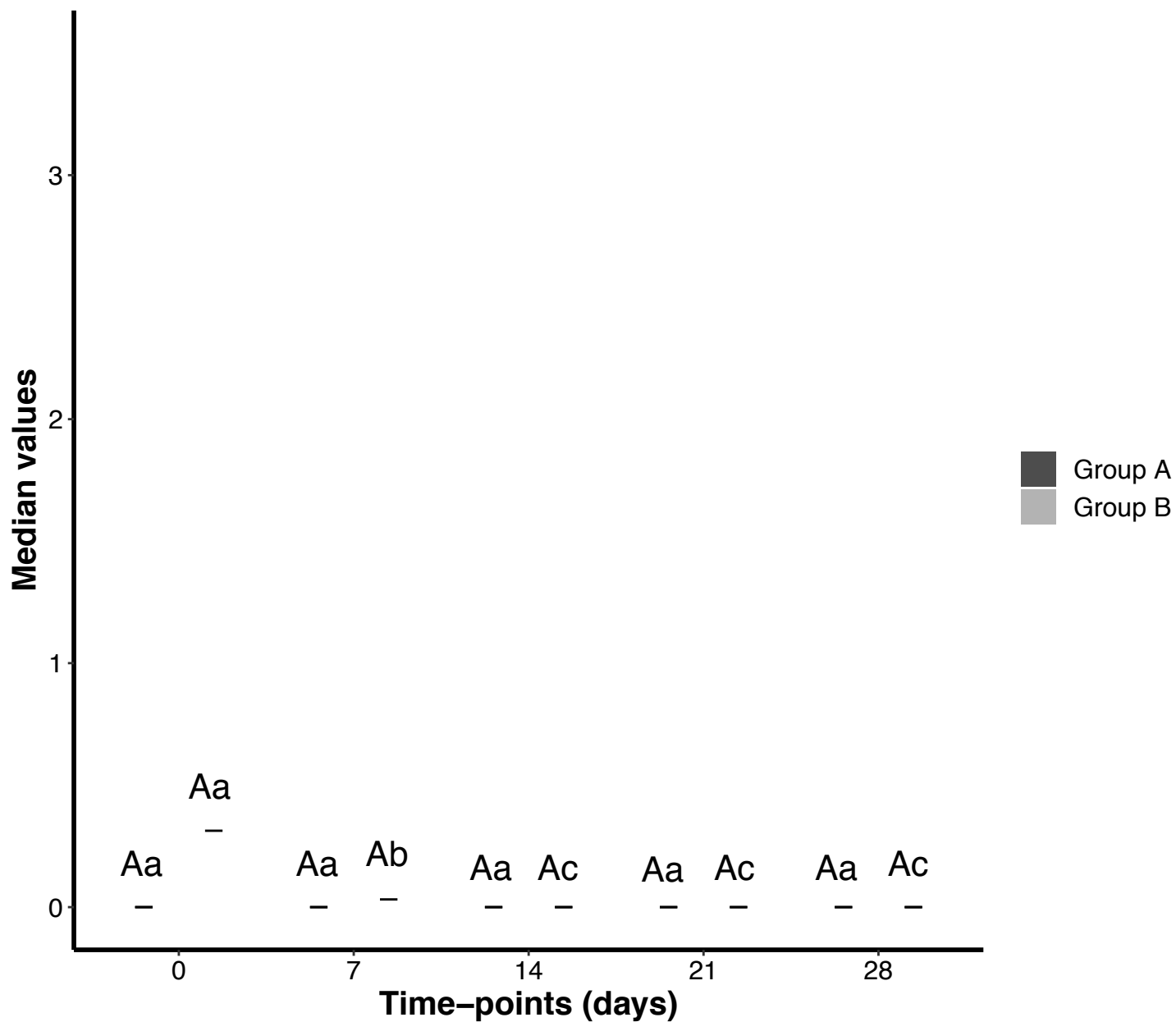

Supplement: Supplementary file 1 [file pathogens-14-01112-s001.zip › Supplementary File S9.pdf]
